# DBI Mediates the Progression of Ankylosing Spondylitis by Regulating CD56^dim^ NK Cells Cytotoxicity Function

**DOI:** 10.1155/ancp/3279688

**Published:** 2025-11-28

**Authors:** Runhan Zhao, Yanran Huang, Xiao Qu, Jun Zhang, Dagang Tang, Zhou Xie, Xiaoji Luo, Zefang Li, Ningdao Li

**Affiliations:** ^1^ Department of Orthopaedic Surgery, Chongqing Municipal Health Commission Key Laboratory of Musculoskeletal Regeneration and Translational Medicine/Orthopaedic Research Laboratory, The First Affiliated Hospital of Chongqing Medical University, Yuzhong, Chongqing, China, cqmu.edu.cn; ^2^ Department of Orthopedics, The First Affiliated Hospital of Chongqing Medical and Pharmaceutical College, Nanan, Chongqing, China; ^3^ Department of Orthopedics, Chongqing University Qianjiang Hospital/Qianjiang Central Hospital of Chongqing, Chongqing, China

**Keywords:** ankylosing spondylitis, druggable gene, machine learning, Mendelian randomization, WGCNA

## Abstract

**Background:**

Ankylosing spondylitis (AS) is an autoimmune disease characterized by low back stiffness and pain, with no cure and a dearth of therapeutic targets.

**Methods:**

Identifying novel AS targets from a list of 5884 druggable genes using weighted gene coexpression network analysis (WGCNA), machine learning, and Mendelian randomization analysis. Investigating the biological functions of the targets through comprehensive bio‐functional analyses; exploring immune‐related functions of the targets based on single‐cell analyses; developing a reliable AS risk prediction model based on the identified targets and clinical data; conducting target drug prediction and molecular docking based on the Enrichr database and the LeDock software.

**Results:**

A novel AS target, diazepam binding inhibitor (DBI), was identified from among 5884 druggable genes. Bio‐functional enrichment analysis indicated that this gene plays a key role in AS by modulating lipid metabolism disorders. Furthermore, single‐cell analysis revealed that the gene likely influences the onset or progression of AS by impairing the cytotoxic function of CD56^dim^ natural killer (NK) cells. Finally, a reliable AS risk prediction model was developed using DBI and clinical data, and several potential therapeutic compounds were identified.

**Conclusion:**

In this study, a novel therapeutic target for AS was identified using multiple algorithms, and it was found to be involved in lipid metabolism and cytotoxic function of CD56^dim^ NK cells. Additionally, a reliable prediction model was developed, and potential therapeutic compounds were identified. In conclusion, this study presents a novel approach for AS treatment.

## 1. Introduction

Ankylosing spondylitis (AS) is an autoimmune disease characterized by low back stiffness and pain, with a prevalence ranging from ~0.07% to 0.32% [1, 2]. The early symptoms of AS are typically mild, characterized by morning back stiffness and intermittent back pain. However, as the disease progresses, AS symptoms typically evolve into persistent severe nocturnal pain, significantly impacting patients’ quality of life [3]. Concurrently, chronic inflammation’s long‐term stimulation results in spinal bony bridges formation and ligament ossification, eventually manifesting as typical spinal “bamboo‐like” changes. In severe cases, it may lead to scoliosis, thoracic movement limitation, and other complications [4]. Therefore, the severe symptoms and incurable status of AS have led to its designation as an “undead cancer.”

The current main treatment modalities for AS are exercise and medication, with the goals of reducing symptoms, improving and maintaining spinal flexibility and normal posture, preserving work capacity, and reducing AS‐related complications being the main treatment goals. Multinational expert groups unanimously recognize that an active exercise program can effectively alleviate symptoms in AS patients [5–7]. Nonsteroidal anti‐inflammatory drugs (NSAIDs), tumor necrosis factor (TNF) inhibitors, and glucocorticoids are commonly used in the treatment of AS, with NSAIDs being the first‐line drugs. TNF inhibitor therapy is typically employed for patients who exhibit ineffectiveness or significant side effects from NSAIDs therapy [3]. Multiple studies demonstrate that five TNF inhibitors (infliximab, etanercept, adalimumab, golimumab, and certolizumab) can rapidly and consistently improve symptoms and spinal function in AS patients [8–11]. However, there are still 40% of patients remaining unresponsive to TNF inhibitor therapy, and glucocorticoids are generally not recommended to be included in the long‐term treatment regimen for AS patients except those with other inflammatory diseases (severe uveitis or inflammatory bowel disease) due to the uncertainty of efficacy and side effects [3]. At this stage, the treatment methods for AS are still insufficient, and patients who do not respond to conventional treatment present a significant challenge for healthcare professionals. Therefore, it is urgent to explore the pathogenesis of AS and to identify new therapeutic targets.

Although the understanding of AS pathogenesis remains incomplete, there is a large number of evidence that immune cells play an important role in its pathogenesis. Previous studies have shown that the number of immune cells (Th17 and Th22) secreting inflammatory cytokines is significantly increased in the peripheral blood of AS patients [12, 13]. Moreover, an increasing number of studies link AS pathogenesis with changes in immune cytotoxicity [14–16]. Among these, the cytotoxic function changes of natural killer (NK) cells have attracted widespread attention. Researchers have found that AS‐related genes are mainly enriched in NK cells and that their cytotoxic function shows significant alterations, while the reduction in CD56^dim^ NK cells and their decreased cytotoxic function may represent underlying pathological mechanisms [17, 18].

With the continuous development of science and technology and the cross‐fertilization of various disciplines, the field of life sciences is demonstrating increasingly robust vitality. Studies have demonstrated that therapeutic targets with genetic evidence are more likely to succeed in clinical trials [19, 20]. Among these, proteins encoded by druggable genes can provide powerful clues for identifying drug targets [21, 22]. Simultaneously, using increasingly mature transcriptome and single‐cell omics technologies, researchers can investigate potential disease targets and explore their related molecular mechanisms, thereby providing new opportunities for disease treatment.

In this study, a novel therapeutic target, diazepam binding inhibitor (DBI), was identified from a list of 5884 druggable genes utilizing weighted gene coexpression network analysis (WGCNA), univariate logistic regression, Mendelian randomization analysis, and machine learning algorithms. Concurrently, transcriptomic and single‐cell data were employed to investigate the molecular mechanisms of DBI in AS. Furthermore, a reliable AS risk prediction model was developed, and multiple potential therapeutic compounds were identified based on DBI and clinical data. Collectively, our study provides novel insights for targeted AS therapy.

## 2. Materials and Methods

### 2.1. Data Collection

The study integrated multiple data sources for comprehensive analysis. A total of 5884 druggable genes were obtained from the Drug–Gene Interaction Database (DGIdb, https://www.dgidb.org/) and previously published studies (Supporting Information 2: File S1) [22, 23]. The GSE73754 dataset, containing peripheral blood transcriptome data from 52 AS patients and 20 healthy controls, was acquired from the Gene Expression Omnibus (GEO, https://www.ncbi.nlm.nih.gov/geo/) database. The genome‐wide association studies (GWAS) data of cis‐expression quantitative trait loci (cis‐eQTL, exposure data) were, respectively, obtained from the eQTLGen Consortium (https://eqtlgen.org/) database and FinnGen Release 9 (https://www.finngen.fi/en) database for Mendelian randomization analysis, aiming to validate the biological importance of key genes in AS. Single‐cell sequencing data of peripheral blood mononuclear cells (PBMCs) from patients and controls were acquired from the Sequence Read Archive (SRA, https://www.ncbi.nlm.nih.gov/sra) database to investigate immune‐related mechanisms of key genes. Three‐dimensional molecular structure data of key genes and targeted compounds were retrieved from the RCSB Protein Data Bank (RCSB‐PDB, https://www.rcsb.org/) and PubChem (https://pubchem.ncbi.nlm.nih.gov/) databases, respectively.

### 2.2. PBMCs Extraction and Real‐Time Quantitative PCR (RT‐qPCR)

Following approval by the institutional ethics committee, peripheral blood samples were collected from both healthy controls and AS patients. PBMCs were subsequently isolated, and RT‐qPCR experiments were conducted to validate the accuracy of the current data analysis findings.

The principal PBMCs isolation protocol was performed as follows: Peripheral blood was initially diluted with an equal volume of phosphate‐buffered saline (PBS). An appropriate volume of density gradient separation medium (Solarbio, P8610) was then added to a centrifuge tube, upon which the diluted blood sample was carefully layered above the separation medium. Centrifugation was subsequently performed at 800 × *g* for 30 min at room temperature. Following centrifugation, the lymphocyte layer (the second visible layer) was carefully aspirated. The isolated PBMCs were then washed through sequential PBS additions (10, 5, and 5 mL), with each washing step followed by centrifugation at 250 × *g* for 10 min. Finally, the purified PBMCs were aspirated, stored overnight at −80°C, and subsequently transferred to liquid nitrogen for long‐term preservation.

As for RT‐qPCR, total mRNA was extracted from PBMC using TRIzol (Invitrogen, USA) according to the manufacturer’s instructions. Then, the RNA was reverse transcribed into cDNA using a reverse transcriptase kit (TaKaRa, Japan). Next, real‐time fluorescence quantitative PCR (Bio‐Rad, USA) was used to amplify the expression level of the target genes, and the relative mRNA expression level of the target gene was indicated by the 2^−*ΔΔ* CT^ value of the internal reference (GADPH). The specific primer sequences for target genes are provided in Table 1.

**Table 1:** Primer sequences of target genes.

Gene	Forward	Reverse
DBI	5′‐GGCGACATAAATACAGAACG‐3′	5′‐TTCCAGGCATCCCACTT‐3′
GZMA	5′‐CAGTTGTCGTTTCTCTCCTGC‐3′	5′‐TGCAGTCAACACCCAGTCTTT‐3′
GZMB	5′‐GAAAGTGCGAATCTGACTTACG‐3′	5′‐TTGTTTCGTCCATAGGAGACAA‐3′
PRF1	5′‐GCTATCGTTAGTGCTAGTGGAT‐3′	5′‐ATCTGTCTGATGCGTATCCAAT‐3′
GADPH	5′‐GGCTGCCCAGAACATCAT ‐3′	5′‐ CGGACACATTGGGGGTAG ‐3′

### 2.3. Mendelian Randomization Analysis

Mendelian randomization analysis was performed using the “TwoSampleMR” R package. Initially, following the three major assumptions of Mendelian randomization [24], instrumental single nucleotide polymorphisms (SNPs) in the cis‐eQTL data were screened according to the following criteria: (1) selecting SNPs with false discovery rate (FDR) < 0.05 and a range of ±100 kb as candidate instrumental variables; (2) removing SNPs linkage disequilibrium in the cis‐eQTL using European samples from the 1000 Genomes Project [25], with the thresholds set as: *r* ^2^ < 0.01, kb = 10,000; (3) deleting instrumental SNPs with a statistic *F*‐value < 10 to mitigate bias caused by weak instrumental variables; and (4) excluding SNPs directly associated with AS using the PhenoScanner (http://www.phenoscanner.medschl.cam.ac.uk/) database. Subsequently, Mendelian randomization analyses were conducted. The effects of exposure factors on outcomes were assessed based on the Wald ratio (WR) when the number of instrumental SNPs was only 1, and the effects of exposure factors on outcomes were assessed based on the inverse variance weighting (IVW) method when there were multiple instrumental SNPs. Meanwhile, the results were quality controlled by heterogeneity analysis and horizontal pleiotropy analysis. Additionally, for the analysis results with multiple instrumental SNPs, when the five algorithms (IVW, weighted median, MR Egger, weighted mode, and simple mode) did not in the same direction of effect, the gene was not included in the subsequent analysis.

### 2.4. Key Genes Identification and Importance Evaluation

The WGCNA was performed to identify key gene modules, beginning with the removal of outlier samples based on clustering algorithms. The analysis proceeded with sequential construction of the relationship matrix, adjacency matrix, and topological overlap matrix (TOM), where the TOM dissimilarity measure was used to delineate gene modules. During adjacency matrix construction, an appropriate soft threshold power (*β*) was selected to ensure the resulting gene network met scale‐free network criteria, with a scale‐free fit index (*R* ^2^) exceeding 0.9. Following module identification, the Pearson correlation algorithm was applied to assess associations between module eigengenes and clinical traits, retaining modules demonstrating significant correlations (absolute correlation > 0.4, *p*‐value < 0.05) for subsequent analysis. These selected modules were then cross‐referenced with the predefined list of druggable genes to derive the final set of candidate genes.

Following the identification of candidate genes, univariate logistic regression analysis was performed to assess their association with AS. The MR analysis was then conducted using cis‐eQTL data of significant genes and GWAS data of AS to further prioritize biologically relevant candidate genes. To further refine the gene selection, we implemented a two‐stage dimensionality reduction approach employing recursive feature elimination combined with random forest (RFE‐RF) and recursive feature elimination combined with support vector machine (RFE‐SVM) algorithms. These machine learning techniques systematically evaluated the predictive importance of candidate genes, with RFE‐RF leveraging ensemble decision trees and RFE‐SVM utilizing maximum‐margin hyperplane separation. Following independent feature ranking by each algorithm, we performed comparative analysis of the resulting gene rankings to identify consensus key genes demonstrating robust selection across both methodologies. This integrative approach enhanced the reliability of our gene prioritization by mitigating potential biases inherent to any single feature selection method. The RFE‐RF analysis was implemented using the “caret” R package (version 7.0.1), while the RFE‐SVM analysis was conducted using the “e1071” R package (version 1.7.16). Both algorithms employed 10‐fold cross‐validation as the resampling strategy, with all other parameters maintained at their default settings unless otherwise specified.

Finally, the importance of key genes was assessed by correlation analysis, differential expression analysis, receiver operating characteristic (ROC) curve, and confusion matrix.

### 2.5. Exploring the Biological Functions of Key Genes

To elucidate the biological functions of the key genes, we performed a comprehensive series of analyses. Gene Ontology (GO) and Kyoto Encyclopedia of Genes and Genomes (KEGG) pathway analyses were first conducted to characterize the functional annotations of these genes. Subsequently, protein–protein interaction networks were constructed using the GeneMANIA database (https://genemania.org/) to identify functionally associated genes. Gene set enrichment analysis (GSEA) was then performed after stratifying samples into high‐ and low‐expression groups based on median expression levels of key genes, enabling examination of pathway activation patterns. Simultaneously, the degree of immune cell infiltration in samples was calculated using the single‐sample GSEA (ssGSEA) and xCell algorithms, and the correlations between AS, key genes, and immune cells were explored. Finally, the microRNA (miRNA)–mRNA interactions network was predicted through the multiMiR R package, which integrates data from miRTarBase, TarBase, and miRecords databases. Finally, the regulatory relationships between miRNAs and mRNAs were visualized using Cytoscape software (version 3.9.1).

### 2.6. Single‐Cell Analysis of Key Genes

The AS, as an autoimmune disease, exhibits distinct alterations in the immune microenvironment. To elucidate the functional mechanisms of key genes within diverse immune cell populations, we analyzed single‐cell RNA sequencing data derived from human PBMCs. This investigation encompassed comparative assessments of both immune microenvironment characteristics and key gene expression patterns between AS patients and healthy controls across various immune cell subtypes. Following the identification of significant intersample variations in key gene expression within NK cells, we subsequently isolated NK cell populations for more detailed functional characterization.

The two principal NK cell subpopulations, CD56^bright^ and CD56^dim^ NK cells, exhibit distinct functional characteristics: CD56^bright^ NK cells predominantly mediate immunoregulatory functions through cytokine production, whereas CD56^dim^ NK cells primarily execute cytotoxic activity via perforin and granzyme secretion. In this study, the proportion differences between NK cells between AS and normal samples were evaluated based on single‐cell dimensionality reduction clustering analysis; the functional differences between NK cells between AS and normal samples were explored through GSEA analysis; the synergistic expression patterns of key genes and NK cytotoxicity‐related genes were explored based on differential expression analysis and Pearson correlation analysis; additionally, the expression patterns of key genes and cytotoxicity‐related genes during NK cell differentiation trajectories were investigated using the “monocle3” R package; finally, the synergistic changes of key genes and toxic genes in AS samples were verified based on RT‐qPCR.

### 2.7. Exploring the Clinical Applications Value of Key Genes

First, we constructed a nomogram based on key genes and clinical data, and evaluated their performance by ROC curve and calibration curve. Meanwhile, the prediction performance of Nomogram is further evaluated based on 200 times 5‐fold cross‐validation. Subsequently, key gene‐targeted drugs were identified utilizing the Enrichr database (https://maayanlab.cloud/Enrichr/) [26], and molecular docking was conducted for the top five drugs selected based on combined score and absence of obvious toxicities. Here, molecular docking and visualization were implemented based on the Ledock software and Pymol software. Ledock is an excellent molecular docking software with molecular docking algorithms of excellent reliability and accuracy [27].

### 2.8. Statistical Analysis

The main analysis process in this study was implemented based on R (V 4.3.2), assessing between‐group differences based on the Wilcoxon test and correlations between variables based on the Pearson algorithm. Quality control of single‐cell data was performed based on the “Seurat” R package, and low‐quality cells were excluded based on strict criteria (<200 genes/cell, <3 cells/gene, > 10% mitochondrial genes, > 3% ribosomal genes, and <0.1% erythrocyte genes). Additionally, doublet cells were removed based on the “DoubletFinder” R package (V 2.0.3), data were normalized based on cell cycle scores, and batch effects were eliminated based on the “Harmony” R package (V 1.2.0). A *p*‐value < 0.05 was considered statistically significant in this study unless otherwise indicated.

## 3. Results

### 3.1. Key Genes Identification and Importance Evaluation

**Figure 1:**
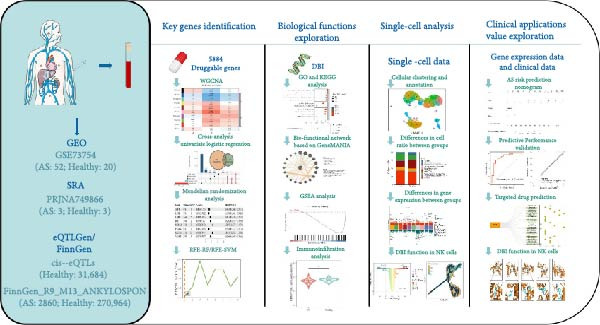
The primary technical route of this study.

The methodological workflow of this study is presented in Figure 1. The identification of key druggable genes was achieved through a multistage analytical approach. Initially, WGCNA was performed on a final cohort of 40 AS patients and 16 healthy controls following quality control and outlier removal (Supporting Information 1: Figure S1A,B). Network construction achieved scale‐free topology (*R* ^2^ ≥ 0.9) at a soft threshold power (*β*) of 11 (Supporting Information 1: Figure S1 C,D). Application of the dynamic tree‐cutting algorithm identified 10 distinct gene modules (Supporting Information 1: Figure S1E). Correlation analyses revealed four modules (blue, turquoise, brown, and pink) demonstrating significant associations with AS status (Pearson correlation > 0.4, *p*  < 0.05) (Figure 2A). Intersection of these significant modules with the druggable gene database yielded 634 candidate genes (Figure 2B). Subsequent univariate logistic regression analysis refined this set to 316 genes meeting statistical significance thresholds (Supporting Information 3: File S2). Mendelian randomization analysis, conducted under stringent selection criteria, identified seven biologically relevant candidate genes (Figure 2C, Supporting Information 1: Figures S2,S3). Final selection through recursive feature elimination with RFE‐RF and RFE‐SVM algorithms established DBI as the primary druggable gene of interest (Figure 2D,E).

Figure 2Key genes identification and importance evaluation: (A) module–trait relationship heatmap; (B) cross‐analysis between druggable genes and key gene modules; (C) Mendelian randomization analysis of candidate key genes; (D, E) identification of key genes based on RFE‐RF and RFE‐SVM; (F) correlation analysis between DBI and clinical traits; (G) analysis of differential expression of DBI between AS and healthy samples ( ^∗∗∗∗^ *p*‐value < 0.0001); (H,I) ROC curve and confusion matrix for assessing the predictive performance of DBI.

**Figure:**
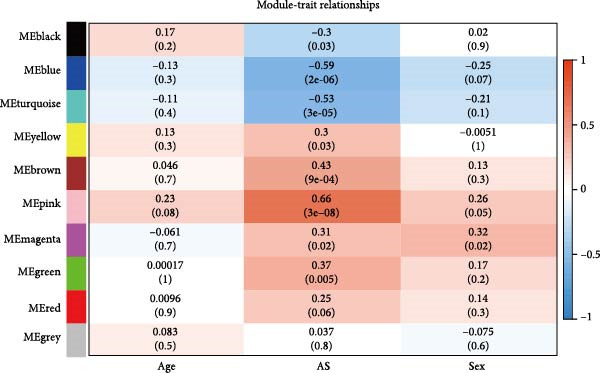
(A)

**Figure:**
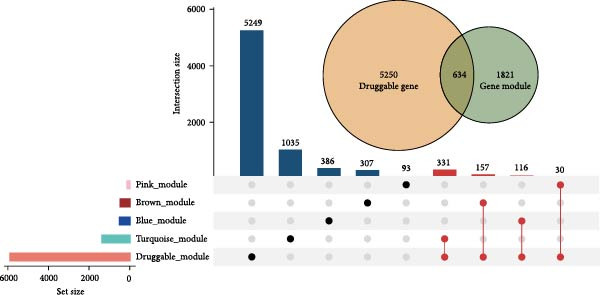
(B)

**Figure:**
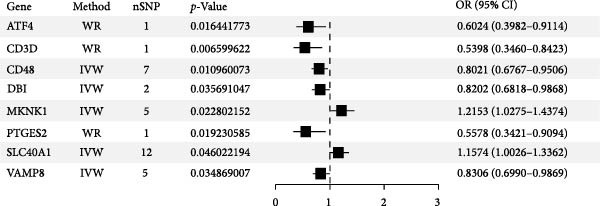
(C)

**Figure:**
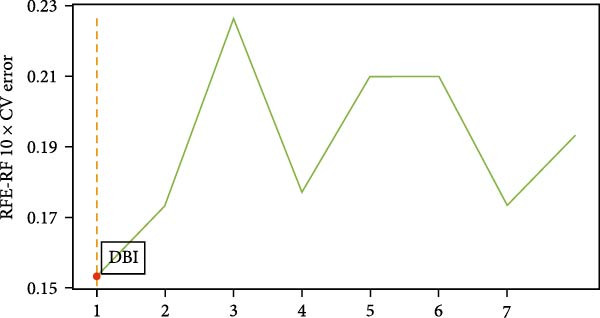
(D)

**Figure:**
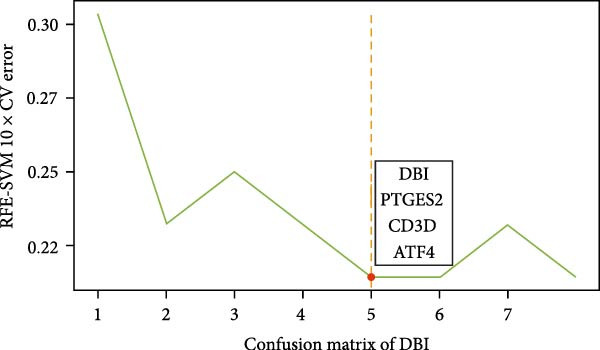
(E)

**Figure:**
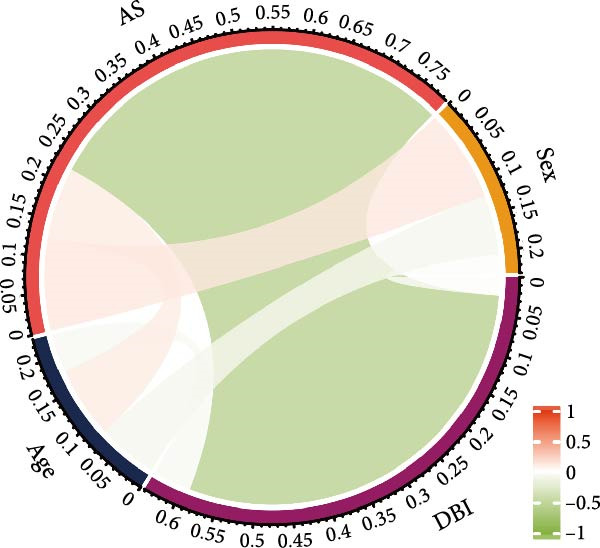
(F)

**Figure:**
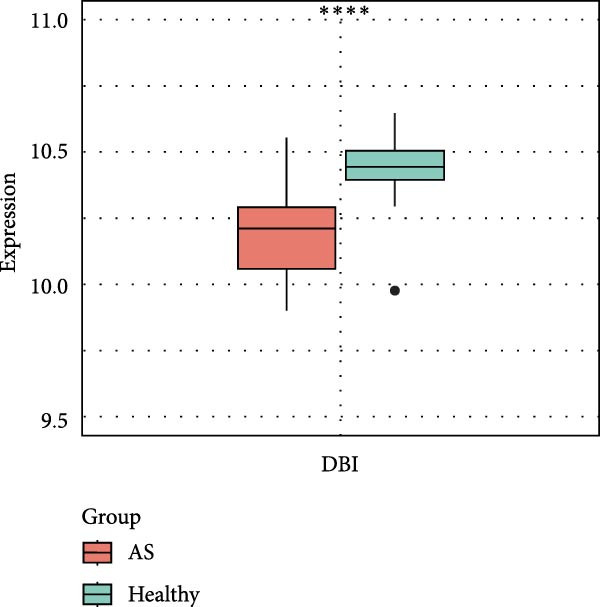
(G)

**Figure:**
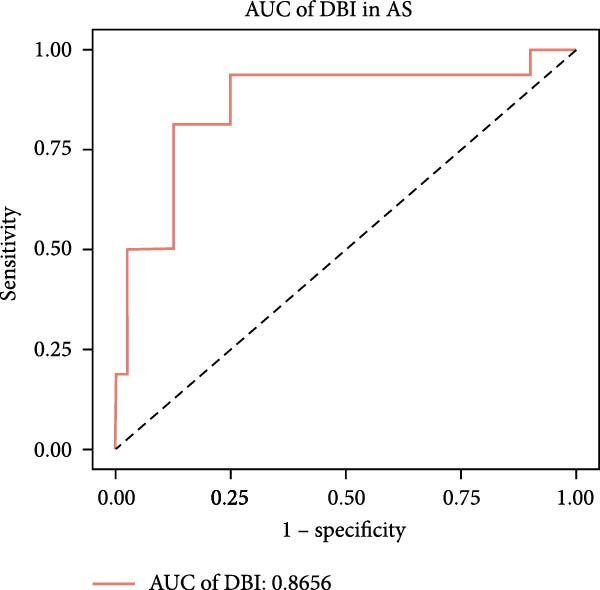
(H)

**Figure:**
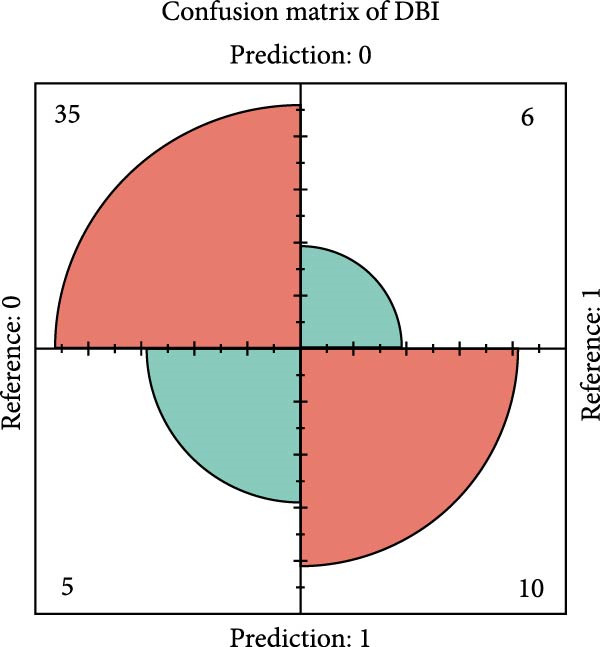
(I)

The functional significance of DBI in AS pathogenesis was systematically evaluated through multiple analytical approaches. Correlation analyses demonstrated a significant negative association between DBI expression and AS status (*p* < 0.05), with this relationship remaining independent of age and gender covariates (Figure 2F,G). Diagnostic performance assessment revealed robust predictive capability, as evidenced by ROC curve analysis (area under the curve [AUC] = 0.8656) and confusion matrix evaluation (Figure 2H,I). Through this comprehensive analytical pipeline employing stringent selection criteria, we identified DBI as the principal druggable gene from an initial pool of 5884 candidates, establishing its critical role in AS pathophysiology.

### 3.2. Exploring the Biological Functions of Key Genes

Functional enrichment analyses revealed DBI’s pivotal involvement in lipid metabolic processes. GO analysis demonstrated significant enrichment of DBI in three biological domains (Figure 3A): (1) biological processes including regulation of CoA‐transferase activity, protein lipidation, and phosphatidylcholine acyl‐chain remodeling; (2) cellular components comprising protein–lipid complexes and endoplasmic reticulum lumen; and (3) molecular functions involving fatty‐acyl‐CoA binding, fatty acid derivative binding, and acyl‐CoA binding. KEGG pathway analysis identified the peroxisome proliferator‐activated receptor (PPAR) signaling pathway as the primary enrichment (Figure 3B), a well‐established regulator of lipid/glucose homeostasis and inflammatory responses [28, 29]. Meanwhile, GeneMANIA network analysis further corroborated DBI’s functional association with lipid metabolism through its interacting partners (Figure 3C). Additionally, GSEA analysis showed that DBI could inhibit the TNF signaling pathway, NF‐kappa B signaling pathway, cytokine–cytokine receptor interaction, osteoclast differentiation signaling pathways, as well as signaling pathways related to NK cell cytotoxicity (Figure 3D), while the first three had been shown to be involved in the pathogenic process of AS [30, 31]. Meanwhile, the DBI‐induced inhibition of osteoclast differentiation may be closely related to osteoporosis caused by AS [32, 33]. Furthermore, based on the immune cell infiltration profiles, we identified distinct immune cell landscapes between normal samples and AS (Supporting Information 1: Figure S4). Notably, NK cells, particularly the CD56^dim^ NK cell subset, were significantly decreased in AS (Figure 3E,F). Concurrently, the expression level of DBI showed a significantly positive correlation with its infiltration level (Figure 3G,H). These findings were highly consistent with the results from the GSEA, collectively indicating that DBI expression and the cytotoxic function of NK cells play important roles in AS. Hence, these findings collectively establish that reduced DBI expression exerts biologically significant effects on multiple pathological processes (especially NK cytotoxic function), driving AS development and progression.

Figure 3Exploring the biological functions of DBI: (A) GO enrichment analysis of DBI; (B) KEGG enrichment analysis of DBI; (C) bio‐functional network of DBI constructed on the basis of GeneMANIA (different colors in each circle correspond to different signaling pathways, and different colors of the connecting lines correspond to different intergenic relationships); (D) GSEA analysis of DBI; (E, F) infiltration differences between NK cells and CD56^dim^ NK cells in AS and normal samples; (G, H) correlation analysis between DBI and NK cells and CD56^dim^ NK cells; (I) mRNA–miRNA regulatory network of DBI.  ^∗∗^ *p* < 0.01 and  ^∗^ *p* < 0.05.

**Figure:**
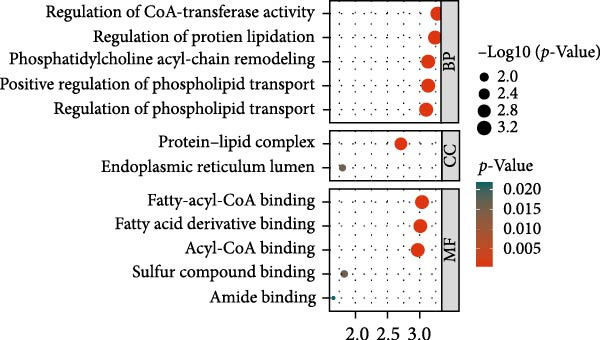
(A)

**Figure:**
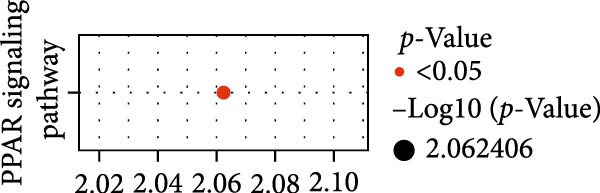
(B)

**Figure:**
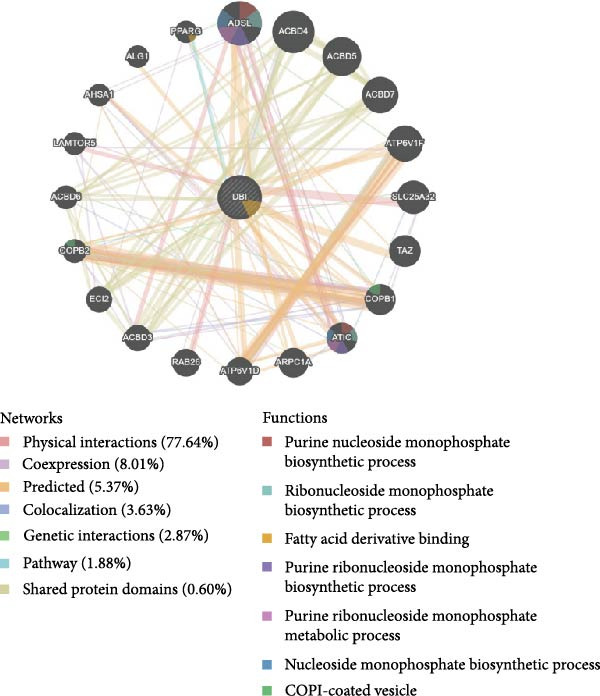
(C)

**Figure:**
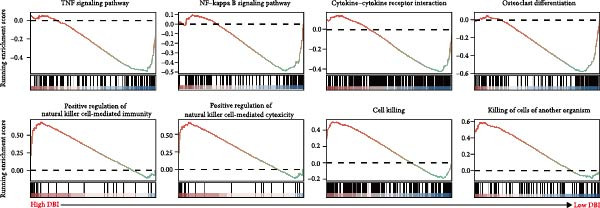
(D)

**Figure:**
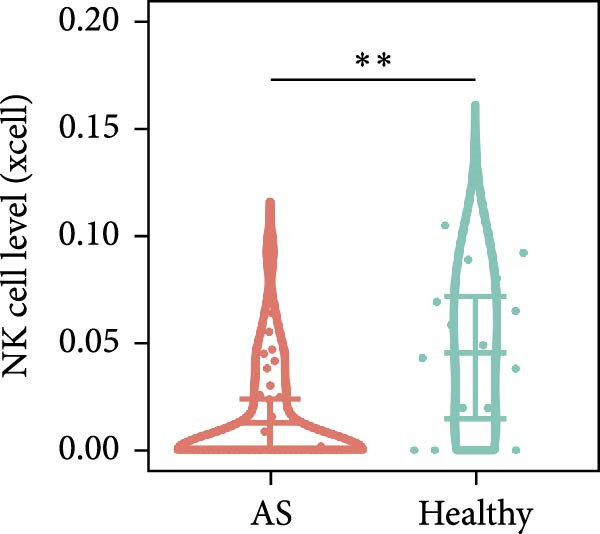
(E)

**Figure:**
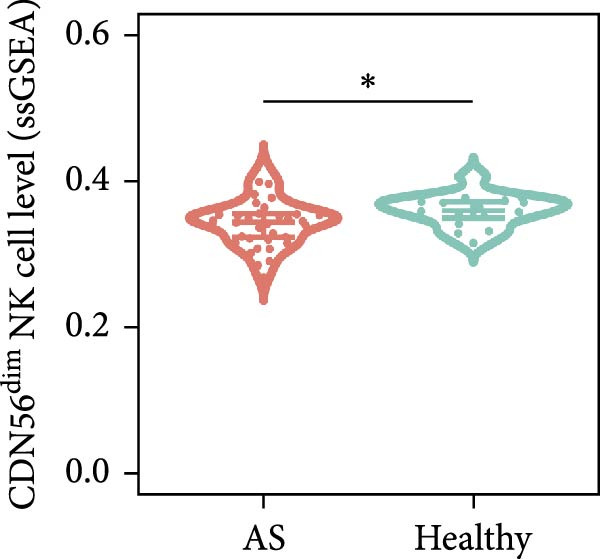
(F)

**Figure:**
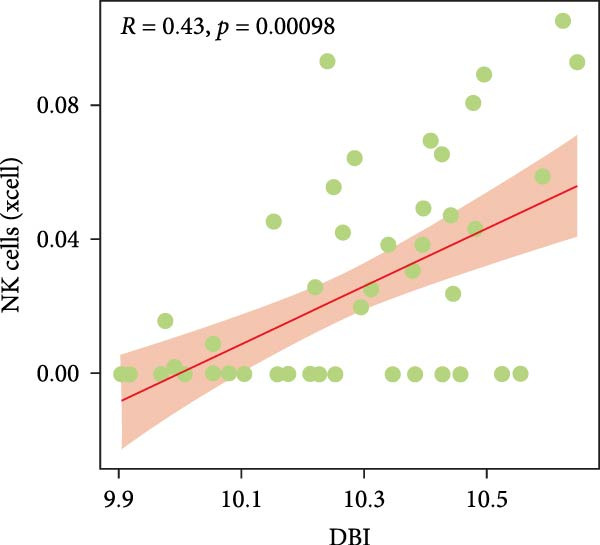
(G)

**Figure:**
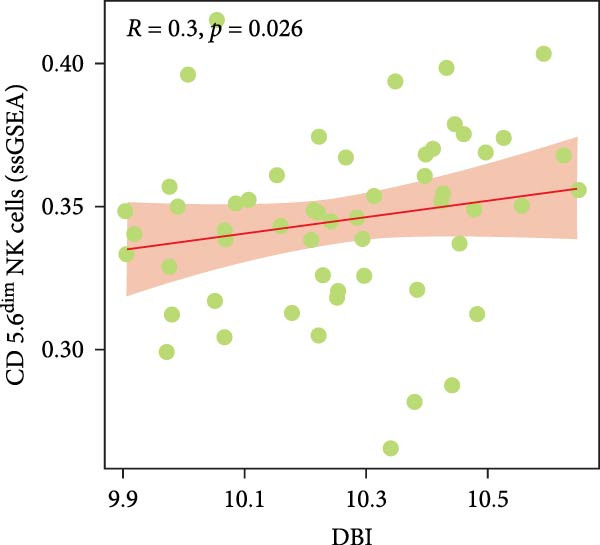
(H)

**Figure:**
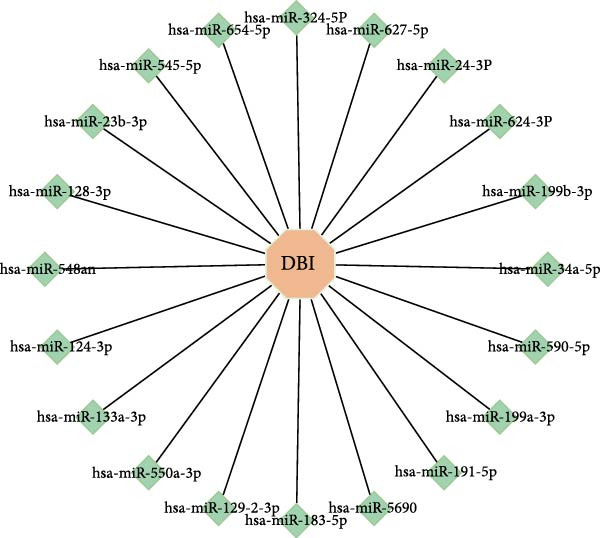
(I)

The miRNAs constitute an evolutionarily conserved class of noncoding RNAs that posttranscriptionally regulate gene expression in eukaryotes through sequence‐specific binding to target mRNAs, resulting in translational inhibition or transcript degradation [34, 35]. Utilizing the “multiMiR” R package, we identified 20 miRNAs exhibiting predicted binding to DBI mRNA and constructed their regulatory network (Figure 3I). These findings suggest that DBI expression may be coordinately regulated by multiple miRNAs, potentially representing a mechanistic contributor to AS pathogenesis. However, the biological significance and functional interplay within this complex regulatory network require further experimental validation through comprehensive follow‐up studies.

### 3.3. Single‐Cell Analysis of Key Genes

Based on the “Seurat” R package, we conducted dimensionality reduction clustering analysis on PBMC single‐cell data from AS and healthy samples, and finally obtained 20 cell clusters (Figure 4A). By observing the expression of cell marker genes in different clusters (Figure 4B), we finally identified 12 types of PMBC cells (Figure 4C). It is worth noting that we observed a significant decrease in the proportion of NK cells in AS samples compared to healthy samples (Figure 4C), which is exactly consistent with the previous results. At the same time, we found that the expression of DBI was significantly enriched in NK cells (Figure 4D). Moreover, the expression level of DBI was significantly decreased in NK cells of AS samples, and this phenomenon was also confirmed in differential expression analysis (Figure 4E,F).

Figure 4Immune‐related analysis of DBI: (A) TSNE plot of preliminary dimensionality reduction clustering of single‐cell data; (B) expression level of immune cell marker genes in different clusters; (C) TSNE plot of the annotated single‐cell data and differences in proportions of immune cells between different samples; (D) TSNE plot of DBI expression in different immune cells; (E) differential gene expression analysis of different immune cells between AS and healthy samples; (F) TSNE plot of DBI differential expression in NK cells between AS and healthy samples.

**Figure:**
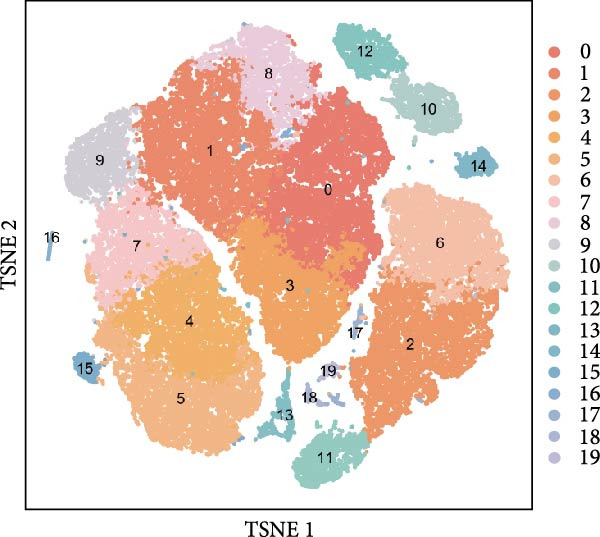
(A)

**Figure:**
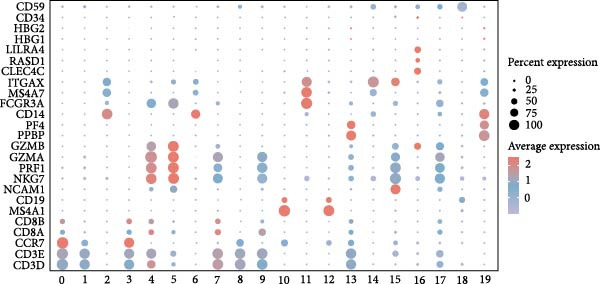
(B)

**Figure:**
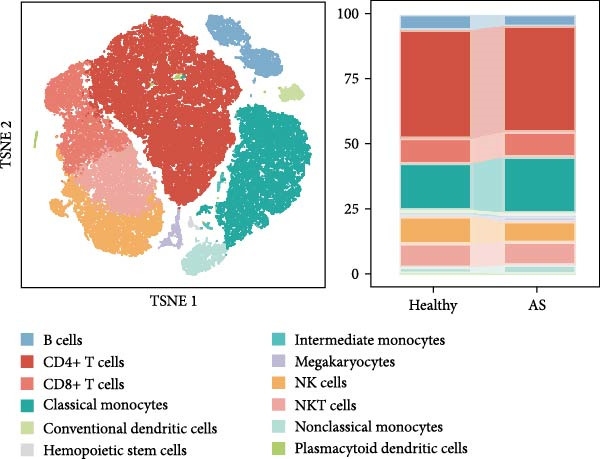
(C)

**Figure:**
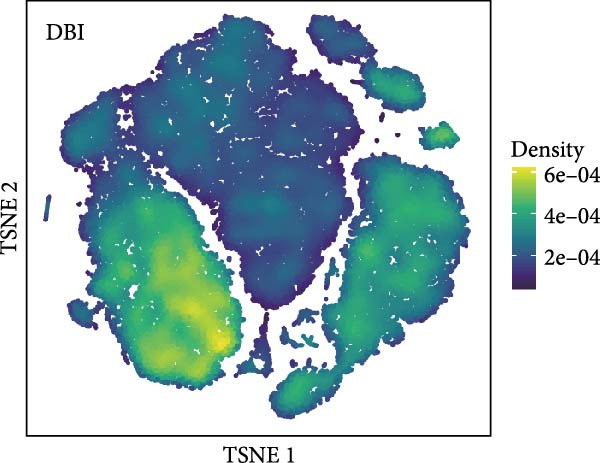
(D)

**Figure:**
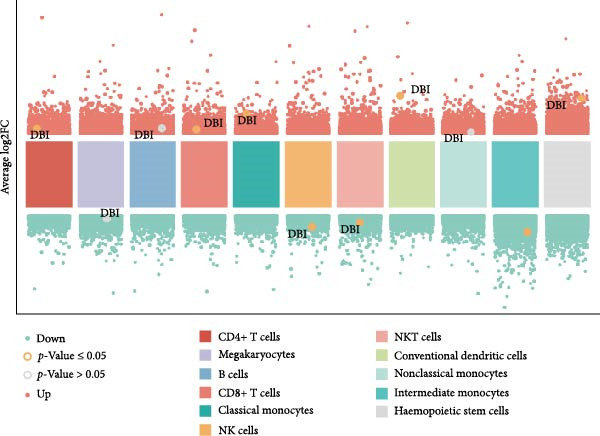
(E)

**Figure:**
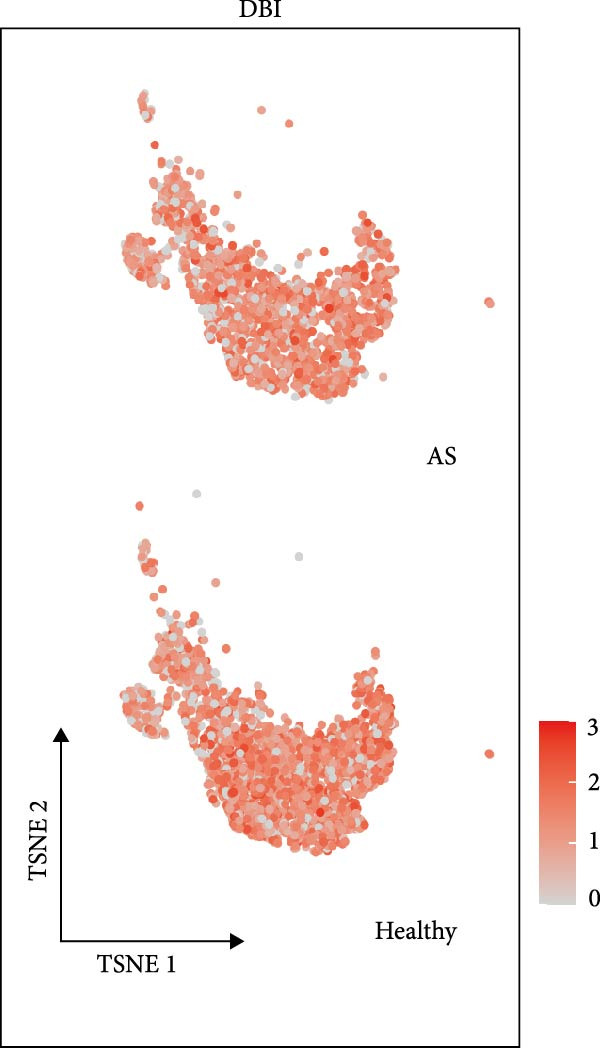
(F)

Combined with previous results, we demonstrated that NK cell levels are closely correlated with AS, while DBI likely contributes to AS pathogenesis or progression by influencing NK cell cytotoxic function.

### 3.4. Exploring the Functions of Key Genes in NK Cells

To elucidate DBI’s functional role in NK cells, we isolated the NK cell population from annotated single‐cell data and performed secondary clustering, identifying nine distinct subclusters (Figure 5A). The aim of this study was to explore the biological function of DBI in conventional NK cells. Therefore, we only utilized the expression levels of CD56 (NCAM1) and CD16 (FCGR3A) to identify CD56^dim^/CD56^bright^ NK cells (Figure 5B) [36, 37]. These subpopulations exhibit specialized effector functions—CD56^dim^ NK cells execute cytotoxic activity through perforin and granzyme secretion, while CD56^bright^ NK cells predominantly mediate immunoregulation through cytokine production [38, 39].

Figure 5Exploring the functions of DBI in NK cells: (A) TSNE plot and marker dotplot of NK cells from secondary dimensionality reduction clustering; (B) TSNE plot after secondary cellnote; (C) heatmap of NK cells’ key protein expression in CD56^dim/bright^ NK cells; (D) TSNE plot of CD56^dim/bright^ NK cells in AS and healthy samples, and differences in proportions of CD56^dim/bright^ NK cells between AS and healthy samples; (E, F) the GSEA analysis results of CD56^dim^ NK cells between AS and healthy sample; (G) differential expression volcano plot of DBI, perforin (PRF1) and granzyme (GZMA, GZMB, GZMH) between AS and healthy samples; (H) RT‐qPCR experiments on PBMC samples from patients; (I) NK cell differentiation trajectory; (J) UMAP plot of DBI, perforin (PRF1) and granzyme (GZMA, GZMB, GZMH) expression changes during NK cell differentiation; (K) correlation analysis of DBI with perforin (PRF1) and granzyme (GZMA, GZMB, GZMH) in CD56^dim^ NK cells.  ^∗^ *p* < 0.05.

**Figure:**
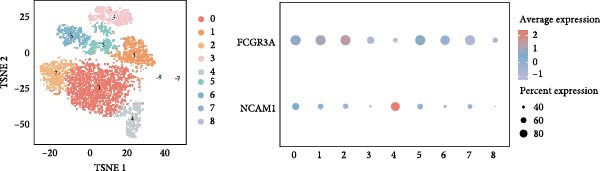
(A)

**Figure:**
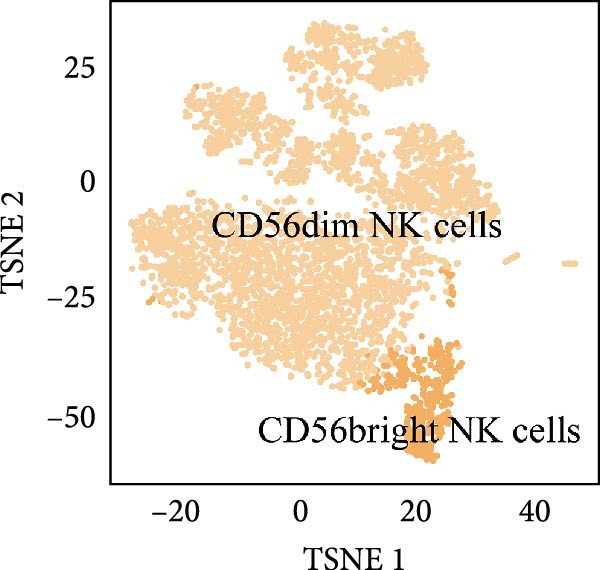
(B)

**Figure:**
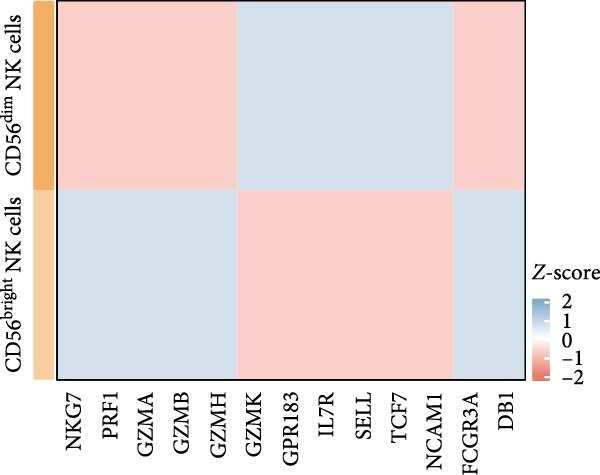
(C)

**Figure:**
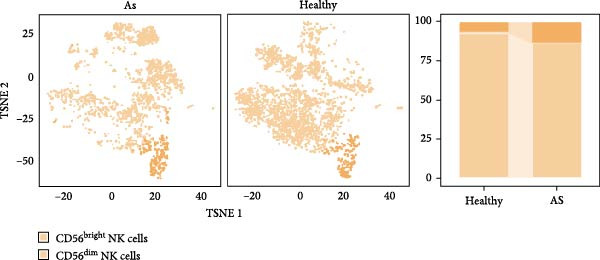
(D)

**Figure:**
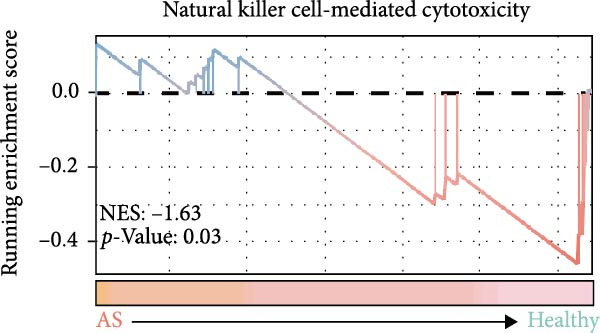
(E)

**Figure:**
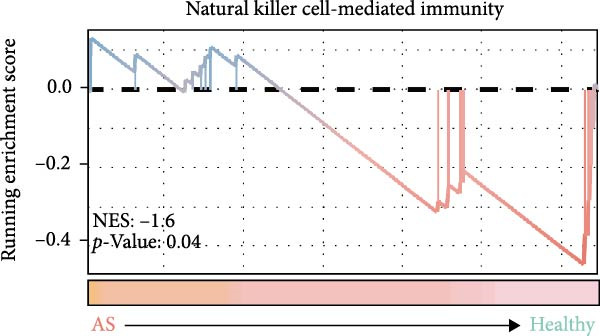
(F)

**Figure:**
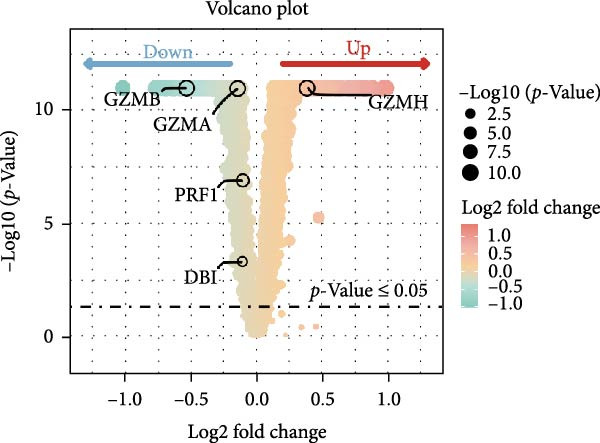
(G)

**Figure:**
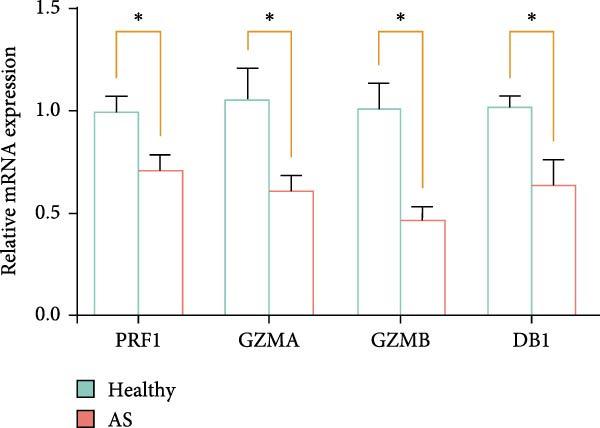
(H)

**Figure:**
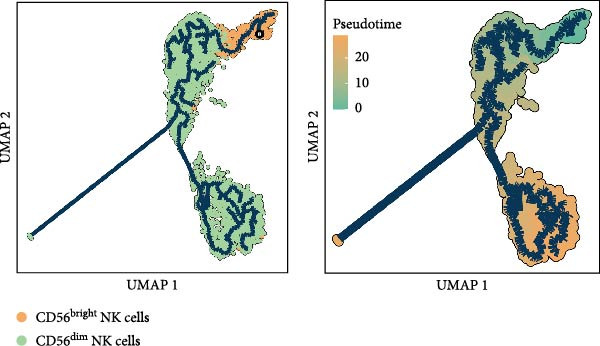
(I)

**Figure:**
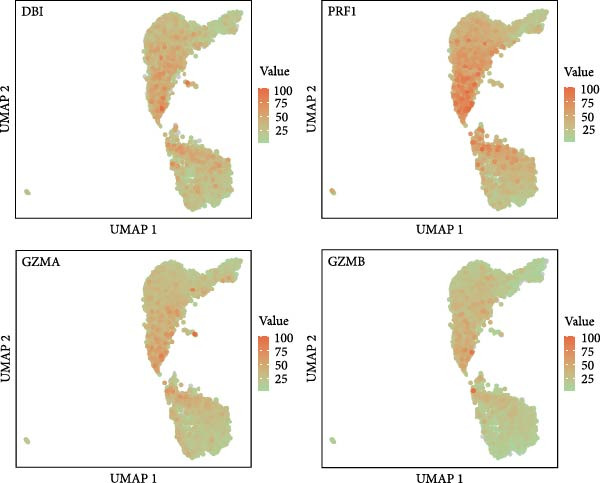
(J)

**Figure:**
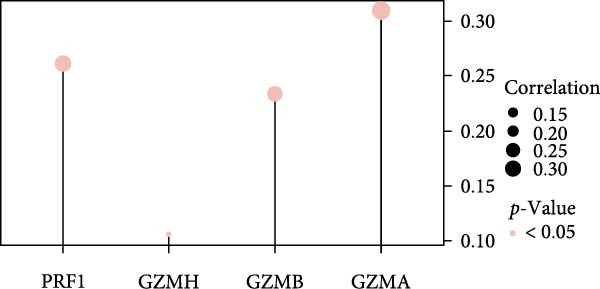
(K)

Transcriptomic analysis revealed distinct functional signatures: CD56^dim^ NK cells exhibited significantly elevated expression of cytotoxic effector molecules (SPON2, NKG7, Granzyme A [GZMA], Granzyme B [GZMB], GZMH), while CD56^bright^ NK cells showed predominant expression of immunoregulatory genes (GPR183, IL7R, SELL, TCF7) and GZMK (Figure 5C), consistent with established literature [40, 41], thereby validating our clustering approach. Notably, DBI demonstrated high expression in CD56^dim^ NK cells (Figure 5C), coinciding with a significant reduction in this cytotoxic subset among AS patients compared to healthy controls (Figure 5D). The decreased percentage of CD56^dim^ NK cells in AS and the significant enrichment of DBI in these cells suggested that DBI likely mediated the pathogenesis or progression of AS by affecting the toxic function of CD56^dim^ NK cells. Therefore, we performed an in‐depth analysis.

Based on GSEA analysis, we found that the toxic function of CD56^dim^ NK cells in AS patients was significantly inhibited (Figure 5E,F); the expression levels of DBI, Perforin 1 (PRF1), GZMB, and GZMA were significantly reduced in AS samples compared to healthy samples (Figure 5G), and this phenomenon was also confirmed in RT‐qPCR experiments of PBMC samples from AS patients (Figure 5H). Additionally, pseudotemporal trajectory analysis revealed the differentiation pattern of CD56^dim^ and CD56^bright^ NK cells, validating CD56^bright^ NK cells as the precursor population to CD56^dim^ NK cells (Figure 5I), which aligns with established literature [42–44]. Furthermore, expression levels of both DBI and cytotoxicity‐related genes progressively increased along the NK cell differentiation trajectories and exhibited a significantly positive correlation (Figure 5J,K). These collective findings indicate that restricted expression of DBI impairs the forward differentiation of CD56^dim^ NK cells, leading to diminished expression of cytotoxicity‐related genes. A previous study indicated a significant correlation between decreased GZMB expression in NK cells and the activity level of AS [18]. Therefore, elucidating the molecular mechanisms by which DBI influences NK cell differentiation and developing targeted therapeutics holds promise as a novel therapeutic strategy for AS.

### 3.5. Exploring the Clinical Applications Value of Key Genes

Utilizing DBI expression data and clinical parameters from the GSE73754 dataset, we developed a predictive nomogram for AS risk assessment (Figure 6A). The model demonstrated robust predictive accuracy, as evidenced by ROC curve analysis (AUC = 0.8781) and calibration curve evaluation (Figure 6B,C). This performance was further validated through 200 iterations of five‐fold cross‐validation, which yielded consistently high AUC and *C*‐index values (Supporting Information 1: Figure S5). Through the Enrichr database, we identified 25 pharmacological compounds predicted to upregulate DBI expression (Figure 6D). Following stringent screening criteria, we selected the top five candidates based on combined enrichment scores and favorable safety profiles for molecular docking analysis (Supporting Information 4: File S3). The results revealed successful binding of all five compounds within the structural pockets of the DBI protein (Figure 6E), suggesting their potential therapeutic applicability for AS. However, comprehensive toxicological and pharmacological assessments remain essential to evaluate their clinical value.

Figure 6Exploring the clinical applications value of DBI: (A) AS risk prediction nomogram; (B, C) ROC curve and calibration curve of the nomogram; (D) predicted target drugs for DBI (which are all overexpressing DBI); (E) molecular simulation docking plots of DBI with the top five target drugs without significant toxicities and with top combined scores.

**Figure:**
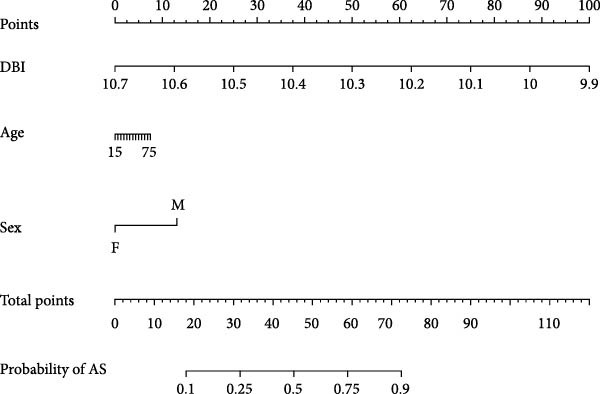
(A)

**Figure:**
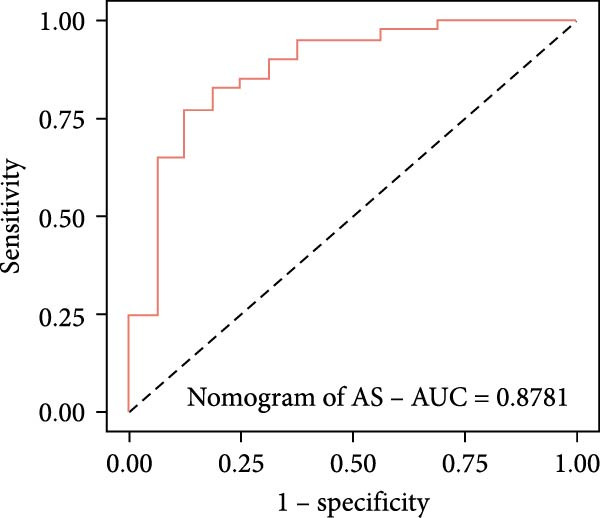
(B)

**Figure:**
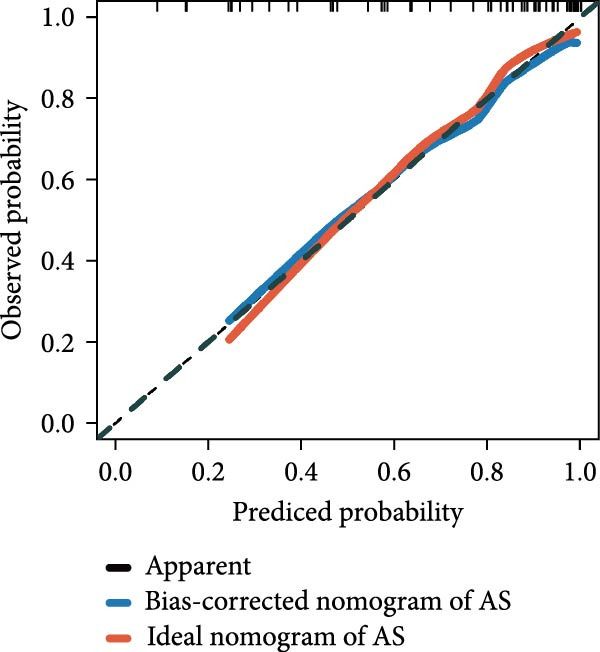
(C)

**Figure:**
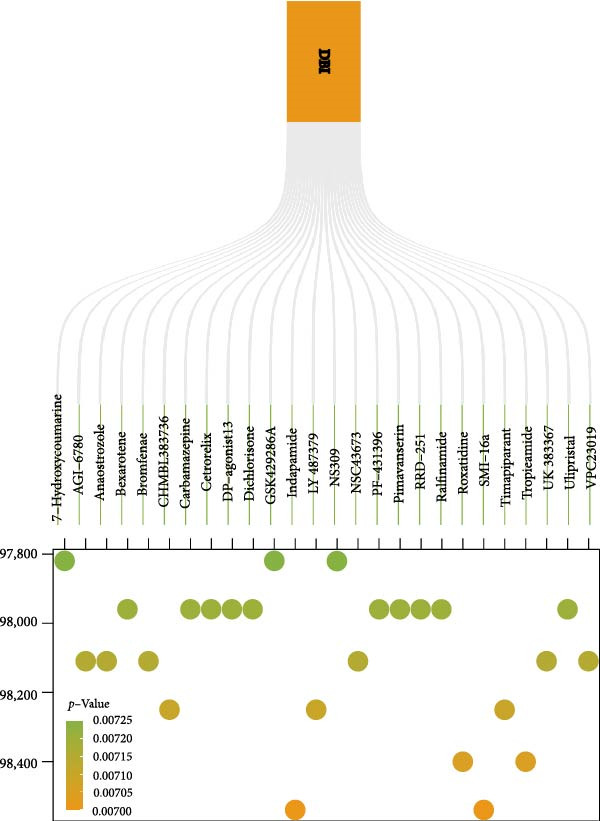
(D)

**Figure:**
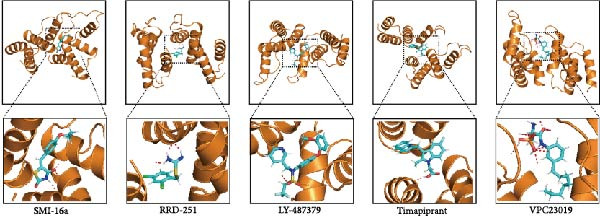
(E)

## 4. Discussion

The AS, as a refractory autoimmune disorder, presents significant therapeutic challenges in contemporary clinical practice. This study systematically identified DBI as a novel therapeutic target from an initial pool of 5884 druggable genes through comprehensive multimodal analysis. The validation analysis showed that the target has excellent predictive performance and is independent of age and gender factors, which makes it highly generalizable in individualized therapy.

DBI/Acyl‐CoA binding protein (ACBP) represents a genetically encoded protein that critically regulates fatty acid transport through PPAR signaling pathways. Our functional enrichment analyses demonstrated DBI’s predominant involvement in lipid metabolic processes via PPAR‐mediated mechanisms, consistent with established literature [45, 46]. PPARs are members of the nuclear hormone receptor superfamily with three isoforms (PPAR*α*, PPAR*β*/*δ*, and PPAR*γ*), which are key transcription factors activated by aptamers, and their mediated PPAR signaling pathway exerts biological effects in the regulation of lipid metabolism, glucose metabolism, and inflammatory response [47, 48]. Extensive research has established that PPAR*γ* demonstrates significant anti‐inflammatory and anti‐fibrotic properties across multiple disease models, with its agonists exhibiting protective effects in various autoimmune conditions [49]. Our findings reveal a marked reduction in DBI expression within PBMCs of AS patients, suggesting a potential disruption of PPAR signaling that may contribute to AS pathogenesis. Meanwhile, the results of GSEA analysis also indicated that DBI has the ability to inhibit the TNF signaling pathway, NF‐kappa B signaling pathway, cytokine–cytokine receptor interaction and the osteoclast differentiation and other signaling pathways, the first three of which have been shown to play key roles in the pathogenic process of AS [30], and the activation of osteoclast differentiation due to decreased DBI expression is likely to be closely related to osteoporosis caused by AS, which is worthy of in‐depth study. These findings collectively provide robust biological evidence supporting DBI’s involvement in AS disease mechanisms through modulation of both inflammatory and metabolic pathways.

Our integrated analyses also revealed that low expression of DBI suppresses NK cell‐mediated cytotoxicity, with this gene exhibiting significant enrichment specifically in the CD56^dim^ NK cell subset. Through pseudotemporal trajectory analysis and correlation studies, we confirmed that DBI plays a critical role in CD56^dim^ NK cell differentiation and demonstrated a strong positive correlation with cytotoxicity‐associated genes (PRF1, GZMA, and GZMB). CD56^dim^ NK cells represent a crucial immunoregulatory population that mediates target cell elimination through perforin and granzyme secretion [38, 39]. Clinical observations have consistently documented significant reductions in CD56^dim^ NK cell frequencies in both AS and systemic lupus erythematosus (SLE), with these alterations correlating with disease activity levels [18, 50, 51]. A large amount of evidence showed that killer cell immunoglobulin‐like receptors (KIRs) and their corresponding specific HLAC ligands are involved in the pathogenesis of a variety of autoimmune diseases by regulating NK cells and T cells functions [52, 53]. Notably, Jiao et al. reported elevated frequencies of KIR2DL1 and KIR2DL5 genes in AS patients compared to healthy controls, suggesting potential NK cells functional impairment that may contribute to disease progression [52]. Moreover, our transcriptomic data and single‐cell analyses demonstrated significantly reduced proportions of CD56^dim^ NK cells in AS samples, validating the accuracy and robustness of our findings.

GZMB, the most extensively characterized member of the granzyme family, plays pivotal roles in tissue repair, chronic inflammation, and immune regulation [54–57]. Multiple studies have documented GZMB downregulation in AS, SLE, and systemic sclerosis, concomitant with impaired cytotoxic function of CD56^dim^ NK cells [18, 50, 58]. Notably, a strong positive correlation has been established between reduced GZMB expression and disease activity in AS [18], underscoring the critical association between CD56^dim^ NK cell dysfunction and AS pathogenesis. And our study showed that DBI was significantly and positively correlated with two granzyme enzymes (GZMA and GZMB) as well as PRF1, and the expression levels of these genes were all decreased in CD56^dim^ NK cells from AS patients. Therefore, these comprehensive research results strongly indicate that the restricted expression of DBI leads to a decrease in the proportion of CD56^dim^ NK cells and impaired cytotoxic functions, which is a key factor in the occurrence or progression of AS.

In summary, DBI, as a critical intracellular regulator of lipid metabolism, profoundly influences the cytotoxic function of NK cells through its core role as an ACBP [46]. Specifically, CD56^dim^ NK cells require substantial energy and biosynthetic precursors upon activation to support chemotaxis and the secretion of cytotoxic granules [59]. DBI serves as a “logistics hub” in this process by efficiently transporting long‐chain acyl‐CoA, which, on one hand, fuels mitochondrial *β*‐oxidation to ensure energy supply, and on the other, provides raw materials for endoplasmic reticulum phospholipid synthesis to maintain membrane structures necessary for cell proliferation and cytotoxic granule secretion [60, 61]. Thus, regular DBI expression and function are foundational for sustaining the efficient metabolic state and potent cytotoxic capabilities of NK cells. Conversely, reduced DBI expression or impaired function leads to metabolic dysregulation, directly weakening NK cell cytotoxicity [62, 63].

At the end of the study, we constructed a clinical AS risk prediction model based on the expression level of DBI and clinical data, and its prediction accuracy and reliability were demonstrated in both the ROC curve and calibration curve. Meanwhile, based on the Enrichr database, we identified the potential target drugs of DBI and performed molecular docking by the Ledock software, an excellent molecular docking software, which is mainly based on the annealing‐genetic crossover algorithm for molecular conformation searching, and presents a strong advantage in terms of both running speed and accuracy [27]. The molecular docking results of this study showed that the small molecule structures of the drugs successfully occupied in the molecular pockets of the proteins, among which SMI‐16a (PIM1/2 Kinase Inhibitor VI) was noteworthy. This drug exerts good efficacy in tumor suppression and osteoclast genesis based on its excellent inhibition of Pim‐1/Pim‐2, and it was a safe and reliable drug, as no significant toxic side effects were found in bone marrow and liver [64, 65]. In this study, we found that low expression of DBI is closely related to AS development and osteoporosis formation, and the function of SMI‐16a overexpression of DBI is likely to have a role in improving AS and inhibiting osteoclast transformation, and these results also provide a mechanistic foundation for developing targeted therapies addressing both the inflammatory and osteoporosis of AS.

### 4.1. Revised Study Limitations

Our study verifies the DBI’s role in NK cell cytotoxicity and AS pathogenesis, but limitations affect its generalizability. The small sample size and single‐ethnicity cohort limit applicability across diverse populations, as genetic and environmental factors may alter DBI function. Technical variability in sequencing methods hinders consistent DBI threshold determination, complicating clinical AS risk prediction. While future technological advances may resolve this, current limitations affect precision, especially in less abundant immune cells. Notably, altered DBI expression in NKT cells and monocytes suggests broader immune roles, potentially impacting cytotoxicity or inflammation, but was underexplored due to our NK cell focus. This may lead to an incomplete understanding of DBI’s immunometabolic effects. Finally, our bioinformatics‐derived hypotheses on DBI deficiency and AS require validation via larger, diverse cohorts, standardized sequencing, and multicell‐type analyses.

## 5. Conclusion

In conclusion, we identified a novel therapeutic target for AS by various advanced algorithms and Mendelian randomization analysis, and found that it plays a great role in the biological process of CD56^dim^ NK cytotoxic dysfunction. Meanwhile, we constructed a reliable prediction model and identified multiple potential target drugs, which provide a novel idea for targeted therapy of AS.

**Nomenclature**
AS: Ankylosing spondylitis
AUC: Area under the curve
cis‐eQTL: cis‐Expression quantitative trait loci
DBI: Diazepam binding inhibitor
FDR: False discovery rate
GO: Gene Ontology
GSEA: Gene set enrichment analysis
GWAS: Genome‐wide association studies
GZMA: Granzyme A
GZMB: Granzyme B
IVW: Inverse variance weighting
KIRs: Killer cell immunoglobulin‐like receptors
KEGG: Kyoto Encyclopedia of Genes and Genomes
NK cells: Natural killer cells
NSAIDs: Nonsteroidal anti‐inflammatory drugs
PRF1: Perforin 1
PBMCs: Peripheral blood mononuclear cells
PPAR: Peroxisome proliferator‐activated receptor
RT‐qPCR: Real‐time quantitative PCR
ROC: Receiver operating characteristic
RFE‐RF: Recursive feature elimination combined with random forest
RFE‐SVM: Recursive feature elimination combined with support vector machine
SNP: Single‐nucleotide polymorphism
ssGSEA: Single‐sample gene set enrichment analysis
SLE: Systemic lupus erythematosus
TNF: Tumor necrosis factor
WR: Wald ratio
WGCNA: Weighted gene coexpression network analysis.

## Ethics Statement

This study was approved by the Clinical Research Ethics Committee of the First Affiliated Hospital of Chongqing Medical University (ID: 2024‐080‐01).

## Consent

The authors have nothing to report.

## Disclosure

All authors read and approved the final manuscript.

## Conflicts of Interest

The authors declare no conflicts of interest.

## Author Contributions

Runhan Zhao, Yanran Huang, and Xiao Qu contributed equally to the work. Runhan Zhao, Yanran Huang, and Xiao Qu performed the conception and design of the study. Ningdao Li, Zefang Li, and Xiaoji Luo guided the methodology section. Runhan Zhao, Yanran Huang, and Xiao Qu performed the data analysis, experimental verification, and visualization. Jun Zhang, Dagang Tang, and Zhou Xie performed the data collection. Runhan Zhao and Yanran Huang wrote the manuscript. Ningdao Li, Zefang Li, and Xiaoji Luo reviewed the manuscript.

## Funding

This research was supported by China Fundamental Research Funds for the Central Universities (Grant 2022CDJYGRH‐019), Chongqing Municipal Technology Innovation and Application Development Specialized Key Projects (Grant CSTB2021TIAD‐KPX0060), and the Doctoral Innovation Project of the First Affiliated Hospital of Chongqing Medical University (Grant CYYY‐BSYJSKYCXXM202446).

## Supporting Information

Additional supporting information can be found online in the Supporting Information section.

## Supporting information


**Supporting Information 1** Figure S1: WGCNA analysis. (A) Sample clustering analysis and outlier removal; (B) Sample clustering dendrogram and trait heatmap after outlier removal; (C, D) Identification and evaluation of the optimal soft threshold (*β*); (E) Gene module clustering dendrogram. Figure S2: Scatter plots for MR analyses of the causal effect of the candidate key genes on AS. Figure S3: Leave‐one‐out analysis of the causal effects of the candidate key genes on AS. Figure S4: Immunoinfiltration analysis. (A) Immuneinfiltration analysis heatmap; (B) Box plot for immune infiltration analysis based on xCELL algorithm; (C) Box plot for immune infiltration analysis based on ssGSEA algorithm. Figure S5: The predictive performance of the nomogram was evaluated based on 200 five‐fold cross‐validation.

**Supporting Information 2** File S1: The list of 5884 druggable genes.

**Supporting Information 3** File S2: 316 genes screened based on univariate logistic regression algorithm.

**Supporting Information 4** File S3: Related information of 26 targeted drugs.

## Data Availability

All raw data in this study can be obtained from the Drug‐Gene Interaction Database (DGIdb, https://www.dgidb.org/), the Gene Expression Omnibus (GEO, https://www.ncbi.nlm.nih.gov/geo/) database, the eQTLGen Consortium (https://eqtlgen.org/) database, the FinnGen Release 9 (https://www.finngen.fi/en) database, the Sequence Read Archive (SRA, https://www.ncbi.nlm.nih.gov/sra) database, the RCSB‐PDB (https://www.rcsb.org/) database, and the PubChem (https://pubchem.ncbi.nlm.nih.gov/) database.
