# Supplementary material for: DBI Mediates the Progression of Ankylosing Spondylitis by Regulating CD56dim NK Cells Cytotoxicity Function
Source: Anal Cell Pathol (Amst). 2025 Nov 28;2025:3279688. doi: 10.1155/ancp/3279688 (PMC12661905; doi:10.1155/ancp/3279688)
Supplement: Supplementary file 1 — Supporting Information 1 Figure S1: WGCNA analysis. (A) Sample clustering analysis and outlier removal; (B) Sample clustering dendrogram and trait heatmap after outlier removal; (C, D) Identification and evaluation of the optimal soft threshold (β); (E) Gene module clustering dendrogram. Figure S2: Scatter plots for MR analyses of the causal effect of the candidate key genes on AS. Figure S3: Leave‐one‐out analysis of the causal effects of the candidate key genes on AS. Figure S4: Immunoinfiltration analysis. (A) Immuneinfiltration analysis heatmap; (B) Box plot for immune infiltration analysis based on xCELL algorithm; (C) Box plot for immune infiltration analysis based on ssGSEA algorithm. Figure S5: The predictive performance of the nomogram was evaluated based on 200 five‐fold cross‐validation. [file ANCP-2025-3279688-s004.zip › Supplementary Figure-File infomation.docx.docx]

**Supplementary Figure 1.** WGCNA analysis. (A) Sample clustering analysis and outlier removal; (B) Sample clustering dendrogram and trait heatmap after outlier removal; (C, D) Identification and evaluation of the optimal soft threshold (β); (E) Gene module clustering dendrogram.

**Supplementary Figure 2.** Scatter plots for MR analyses of the causal effect of the candidate key genes on AS.

**Supplementary Figure 3.** Leave-one-out analysis of the causal effects of the candidate key genes on AS.

**Supplementary Figure 4.** Immunoinfiltration analysis. (A) Immuneinfiltration analysis heatmap; (B) Box plot for immune infiltration analysis based on xCELL algorithm; (C) Box plot for immune infiltration analysis based on ssGSEA algorithm

**Supplementary Figure 5.** The predictive performance of the nomogram was evaluated based on 200 5-fold cross validation.

**Supplementary File 1.** The list of 5884 druggable genes.

**Supplementary File 2.** 316 genes screened based on univariate logistic regression algorithm.

**Supplementary File 3.** Related information of 26 targeted drugs.
